# Analysis of two-gene signatures and related drugs in small-cell lung cancer by bioinformatics

**DOI:** 10.1515/med-2023-0806

**Published:** 2023-10-05

**Authors:** Yi Li, Xiwen Zhou, Zhi Lyu

**Affiliations:** The School of Clinical Medicine, Fujian Medical University, Fuzhou, China; Medical College, Shantou University, Shantou, China; Department of Senior Cadres Ward, Zhongshan Hospital of Xiamen University, School of Medicine, Xiamen University, Xiamen, China

**Keywords:** bioinformatics, biomarkers, small cell lung cancer, individualized diagnosis, targeted drugs

## Abstract

Small-cell lung cancer (SCLC) has a poor prognosis and can be diagnosed with systemic metastases. Nevertheless, the molecular mechanisms underlying the development of SCLC are unclear, requiring further investigation. The current research aims to identify relevant biomarkers and available drugs to treat SCLC. The bioinformatics analysis comprised three Gene Expression Omnibus datasets (including GSE2149507, GSE6044, and GSE30219). Using the limma R package, we discovered differentially expressed genes (DEGs) in the current work. Gene Ontology and Kyoto Encyclopedia of Genes and Genomes analyses were made by adopting the DAVID website. The DEG protein–protein interaction network was built based on the Search Tool for the Retrieval of Interacting Genes/Proteins website and visualized using the CytoHubba plugin in Cytoscape, aiming to screen the top ten hub genes. Quantitative real-time polymerase chain reaction was adopted for verifying the level of the top ten hub genes. Finally, the potential drugs were screened and identified using the QuartataWeb database. Totally 195 upregulated and 167 downregulated DEGs were determined. The ten hub genes were NCAPG, BUB1B, TOP2A, CCNA2, NUSAP1, UBE2C, AURKB, RRM2, CDK1, and KIF11. Ten FDA-approved drugs were screened. Finally, two genes and related drugs screened could be the prospective drug targets for SCLC treatment.

## 1 Introduction

Lung cancer refers to one of the most commonly seen cancers globally, showing high morbidity and mortality rates. Each year, around 2.2 million new cases of lung cancer as well as over 1.8 million lung cancer deaths are reported across the world [1]. Small-cell lung cancer (SCLC) is considered a type of lung cancer. It occupies 15% of all lung cancer-related deaths. Most SCLC patients exhibit systemic metastases at the time of diagnosis. As a result, its 5 year survival rate is around 5% [2,3]. Chemotherapy for SCLC frequently fails because SCLC is drug-resistant, which further deteriorates therapeutic outcomes [4]. On the other hand, for the immune surveillance mechanism of SCLC, although the recent immune insertion point blockers for SCLC patients have brought hope for the treatment of SCLC, it only benefits a small number of SCLC patients, not for most of SCLC patients [5]. Therefore, it is essential to develop efficient diagnostic techniques and treatment strategies for SCLC patients.

High-throughput genome sequencing has enabled significant advancements in the diagnosis and therapy of cancer [6]. Following the analysis of clinical and molecular sequencing data, bioinformatic methods can provide new ideas for understanding cancer development. To date, with the development of bioinformatics, there are many studies on SCLC, not only on target genes [7–9] but also on non-coding RNA (ncRNA) [10], and genome-wide studies on SCLC [11]. Although the current research results have enabled us to further understand the molecular level of SCLC, it is still not effective for studying the biological process of SCLC. The molecular mechanisms of SCLC have not been completely illustrated.

The term “drug repositioning” refers to the process of using an FDA-approved drug to treat a disease or condition that is beyond its current indication [12]. The development of new antineoplastic drugs has stalled because of the high cost and time to market, as well as drug toxicity and therapeutic effects [13]. “Drug repositioning” has inspired the use of novel approaches to cancer treatment [14]. For example, disulfide, a drug used for treating alcoholism, has been discovered to exhibit antitumor activity against non-small cell lung cancer (NSCLC), liver cancer, breast cancer, prostate cancer, pancreatic cancer, glioblastoma, as well as melanoma [15]. Another example is chlorpromazine, a high-dose antipsychotic drug approved by FDA as an antineoplastic drug [16]. Therefore, we hypothesized that FDA-approved drugs could be tested using bioinformatic techniques to develop novel antineoplastic drugs for SCLC.

**Figure 1:**
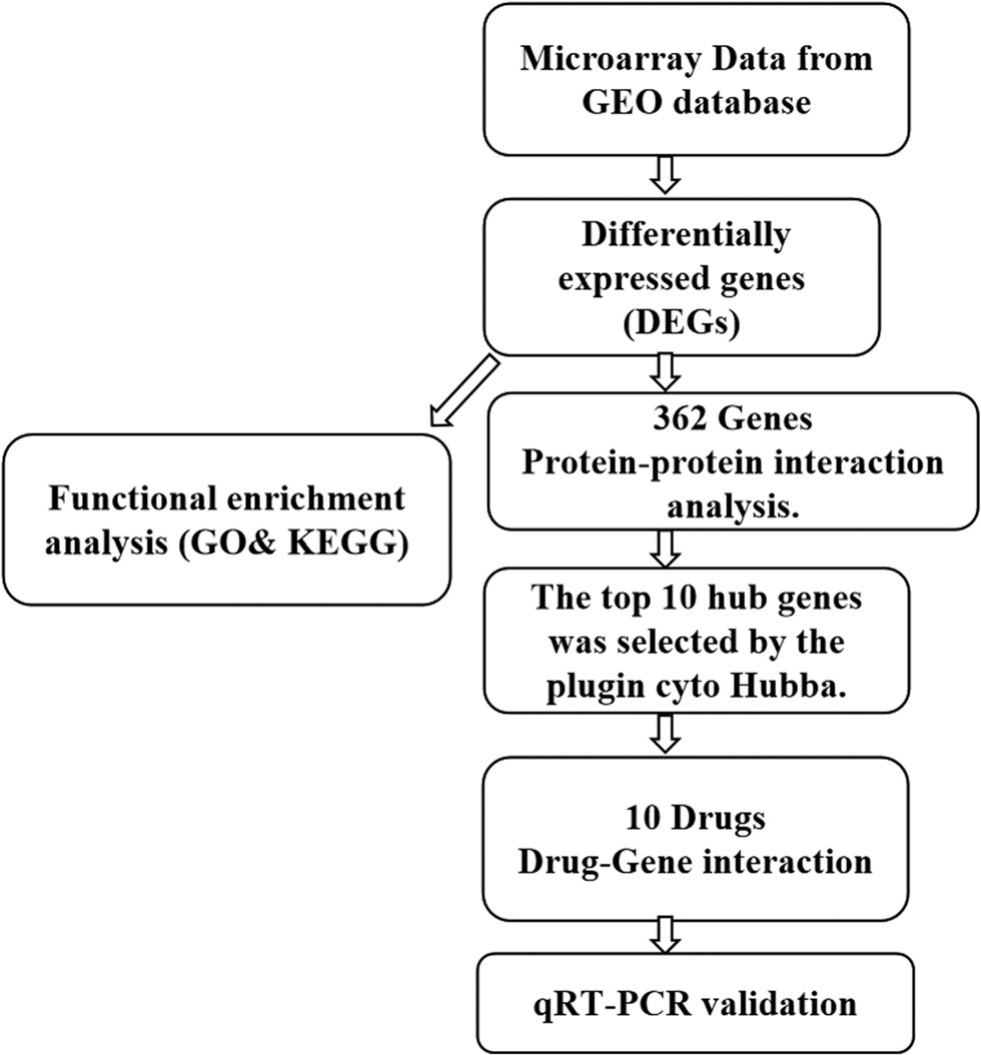
Workflow chart of integrative bioinformatics in this study.

As the molecular regulation is still unknown, the therapeutic effects of drugs are limited. Therefore, it is necessary to detect biomarkers and drugs to treat SCLC. In the current work, bioinformatics analysis was adopted for discovering promising biomarkers and available drugs for SCLC. We selected three microarray datasets from the Gene Expression Omnibus (GEO) database for analysis and also identified differentially expressed genes (DEGs) between the SCLC groups and normal groups. We further performed Gene Ontology (GO) annotation, Kyoto Encyclopedia of Genes and Genomes (KEGG) pathway annotation, as well as protein–protein interaction (PPI) analysis. Finally, the possible biomarkers were identified, and potential drugs related to the treatment of SCLC were screened. Figure 1 presents the workflow of the current study.

## 2 Materials and methods

### 2.1 SCLC dataset

A large amount of gene expression data, such as microarray and high-throughput data, are stored in the GEO database [17]. GSE149507, GSE6044, and GSE30219 were downloaded from the GEO database. In addition, the platform adopted for the microarray dataset was GPL23270 (Affymetrix Human Genome U133 Plus 2.0 Array). GSE149507 includes 36 samples, among which 18 are tumor tissue samples, with 18 being normal tissue samples. GSE6044 includes nine SCLC tissue samples and five normal lung tissue samples. There are 21 lung SCLC samples and 14 non-tumoral lung samples in the GSE30219 dataset.

### 2.2 Identification of DEGs

For the purpose of identifying DEGs, we adopted the limma R package. Genes with |logFC| > 2, and *p*-value < 0.05 were regarded to be DEGs. Genes with downregulated expression in DEGs were assigned logFC < −2, and genes with upregulated expression were assigned logFC > 2. Venn software was used to filter the overlapping DEGs in the three sets of data.

### 2.3 Biological function analysis and pathway enrichment analysis

As an online data analysis website, DAVID (https://david.ncifcrf.gov/) was adopted for performing the GO and KEGG pathway enrichment analysis [18]. Statistical significance was detected at *P* < 0.05.

### 2.4 PPI network construction and selection of hub genes

The STRING database (http://string-db.org) integrates various proteins to construct their interaction networks [19]. In the current work, we created a network of interacting DEGs using STRING software. The interaction network created by DEGs was visualized based on Cytoscape software (http://www.cytoscape.org/) [20]. In addition, the top ten hub genes (scores > 2) were screened with the Hubba plugin in Cytoscape [21].

### 2.5 Screening of existing drugs

In this study, the QuartataWeb (http://quartata.csb.pitt.edu/) integrates, organizes, and displays drug-gene interactions and gene-pharmaceutical information from the stick and drug bank [22]. Through the database and support from previous literature, the top ten hub genes were screened for similarities to existing or failed FDA-approved drugs.

### 2.6 Cell culture

Normal human lung cell line (HLF-a) and human typical SCLC cell line (NCI-H1688) were purchased from Procell (Wuhan, China). Procell offered all cells and their special culture medium. In addition, all the cells were cultivated at 37°C in a humid environment with the concentration of 5% CO_2_ and were exposed to STR profiling.

### 2.7 RNA extraction and quantitative real-time polymerase chain reaction (qRT-PCR)

Using TRIzol reagent, total RNA was isolated from HLF-a and H1688 cells (Invitrogen, CA, USA). By adopting a PrimeScript Reverse Transcriptase Reagent Kit, we performed reverse transcription (RT) of complementary DNA (cDNA) (TakaRa, Tokyo, Japan). In addition, cDNA aliquots were amplified with SYBR Green PCR Master Mix (TaKaRa, Tokyo, Japan). GAPDH acted as an endogenous control. Table 1 presents the sequence of positive and antisense primers involved.

**Table 1:** Primers used for qRT-PCR

Gene	Primer	Sequence 5′–3′
NCAPG	Forward	CTCAGGGGTGTAAAAGCAACCCAAG
	Reverse	ATCACTTTCAGAGTCGGCTTCAGCA
BUB1B	Forward	ATCCTGGCTAACTGTTCTTCTCCCT
	Reverse	TGGCTAAGTTTCCAGAAGGACCCAT
TOP2A	Forward	AATGCTCAGCTCTTTGGCTCGATTG
	Reverse	AATGTACCATTCAGGCTCAACACGC
CCNA2	Forward	ACCAAGAAACAAGTTCTGAGAATGGAGC
	Reverse	AGGCTGCTGATGCAGAAAGTATTGG
NUSAP1	Forward	AGCAAAGGTTTTGGGAATGCGAAGG
	Reverse	TCGTGACTAAAGTGGGGATGACAGC
UBE2C	Forward	GTTCCTGTCTCTCTGCCAACGC
	Reverse	TCATCAGCTCCTGCTGTAGCCTTTT
AURKB	Forward	CGAACAGCCACGATCATGGAGGAG
	Reverse	CTCCCTTGAGCCCTAAGAGCAGATT
RRM2	Forward	TAGGCGAGTATCAGAGGATGGGAGT
	Reverse	CAGCCAAGTAAGGGCACATCTTCAG
CDK1	Forward	TCCTACAGGGGATTGTGTTTTGTCA
	Reverse	AGGTATTCCAAAAGCTCTGGCAAGG
KIF11	Forward	GCGGGGTTCCATTTTTCCAGCATA
	Reverse	GTTGATCTGGGCTCGCAGAGGTAAT

## 3 Results

### 3.1 Identification of DEGs

**Figure 2:**
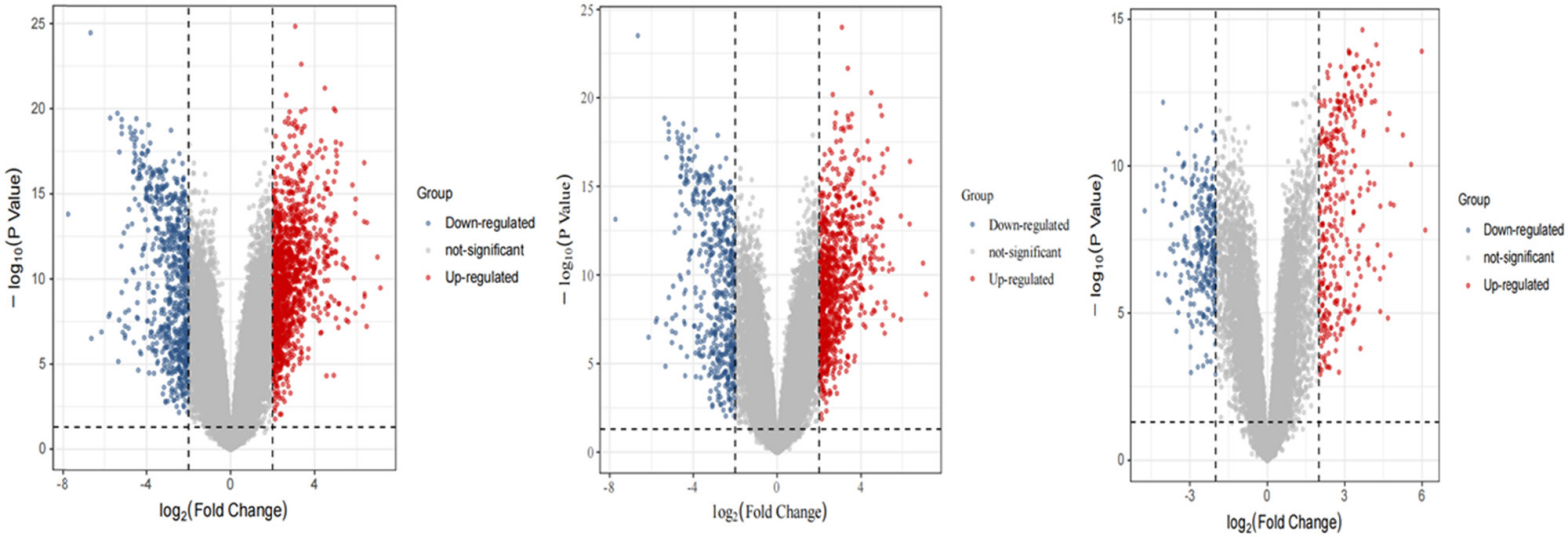
Volcano plot of differentially expressed genes between SCLC tissues and normal lung tissues in datasets GSE6044, GSE30219, and GSE149507. Red denotes genes with high expression in tumor tissues, and blue stands for low expression in tumor tissues. (a) GSE6044; (b) GSE30219; and (c) GSE149507.

**Figure 3:**
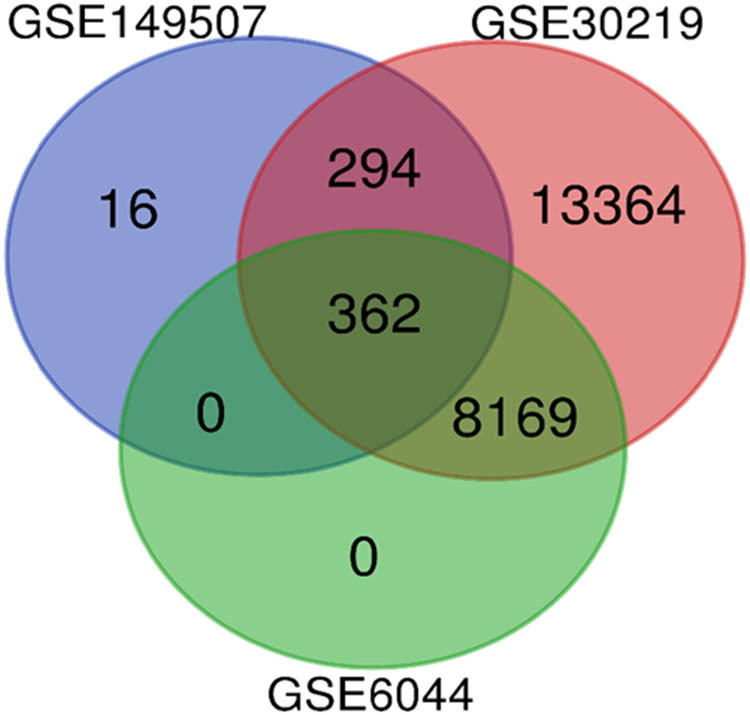
A total of 363 DEGs were found in the three databases (GSE149507, GSE6044, and GSE30219).

The R limma package identified DEGs from the three datasets based on the filtering conditions. There were 22,189 DEGs in GSE30219, 8,563 of which were upregulated and 13,626 of which were downregulated. GSE149507 had 672 DEGs, of which 378 showed upregulation and 294 presented downregulation. GSE6044 has 8,537 DEGs, with 3,874 upregulated and 4,657 downregulated (Figure 2). By intersecting these DEGs using the Venn diagram, 362 overlapping DEGs were acquired, including 195 upregulated genes and 167 downregulated genes (Figure 3, Table 2). Normalization has been performed before obtaining overlapping DEGs.

**Table 2:** Identified DEGs

DEGs*	Gene name
Upregulated	RAB33A, SMC2, TYMS, GNAZ, HMGB3, CDC7, CHEK1, PRAME, TPH1, HRASLS, GMNN, CA9, MB, KIF20A, COL10A1, CTNNA2, ESRRG, HOXB2, CXCL9,DLK1, CDC45, HOXD10, CHRNA5, PAX9, PAX6, RFC4, TFF3, CBFA2T2, ORC1, KCNB2, CAMK2B, PFN2, TROAP, FZD3, ONECUT2, HIST1H2BH, CDK5R1, MYBL2, OLE2, RAD54L, ELOVL4, AMPH, PCSK1N, PTH2R, PROX1, AURKB, AURKA, FEN1, EPHA7, ADGRB2, DSP, CALCA, GREM1, SNAP25, PRDM13, MCM2, CDKN2A, HIST1H2AE, PROM1, ELAVL4, KCNK12,CALB1, LMNB1, ENO2, PCSK2, ELAVL2, FBXO5, UCHL1, CST1, WASF1, KCNA1, BIK, PMAIP1, CDK1, ACTL6B, RNASEH2A, AP3B2, HOXD11, KCNJ6, EYA2, CENPF, ORC6, CRMP1, TRIP13, NPTX1, PSAT1, SCN2A, BIRC5, NRCAM, RACGAP1, ADCYAP1, MNX1, KCNC1, RGS7, DDX25, COCH, ACYP1, CD24, ELAVL3, EEF1A2, SLCO5A1, SALL1, CENPE, KCNMB2, MKI67, STIL, DNAJC12, CDC6, CRYBA2, HMP19, CCNA2, RBFOX1, RPRM, POU3F2, CELSR3, NKX2, HOXA10, GAD1, RAB3B, SCG5, RAD51AP1,HMMR, DLX6, EXO1, GPR19, PTTG1, MAD2L1, PTTG3P, DDC, NEUROD1, KIF5C, NEK2, NDC80, CEL, PCDH8, ADAMDEC1, GHRH, SPOCK1, FOXG1, NCAPG, MAGEA12, SH3GL2, KIF4A, CDKN3, ZWINT, KIF2C, KIF23, UGT8, KIF15, TOP2A, RRM2, COL11A1, SCG2, CALCB, EZH2, DCX, RGS17, CHGB, NUSAP1, SYT1, CDH2,KIF11, BUB1B, PRC1, CCNE2, ESPL1, DLX5, CDC20, UBE2C, GRP, STMN2, CXCL13, TPX2, LHX2, POU4F1, TTK, SOX11, PBK, PCP4, NMU, INA, TAGLN3, CLGN, GNG4, MAGEA6, SCGN, ASCL1, RIPPLY3, ISL1, PCSK1, MMP12, SCG3, NOL4, INSM1, CHGA
Downregulated	WIF1, PPBP, CLDN18, SCGB1A1, CPB2, PLA2G1B, SFTPC, AGER, SFTPD, TNNC1, PGC, AQP4, CYP4B1, CPA3, HPGD, CA3, SLC6A4, FCGR3B, CLIC3, FOLR1, SDPR, SCN7A, ANXA3, C4BPA, CA4, OLR1, TYRP1, CEACAM6, FABP4, SFTPB, PTGS2, ACADL, BMP5, SLPI, FOSB, SCEL, AGTR1, CH25H, GPX3, TCF21, GPRC5A, ZBTB16, HCAR3, GDF10, LYVE1, ADH1B, SELENBP1, CAV1, ADIRF, MMRN1, AOC3, MSLN, FXYD1, GHR, FLRT3, HSD17B6, EMCN, SLC34A2, MRC1, NR3C2, EDNRB, SLC6A14, CFD, FOXF2, HPGDS, RNASE4, SLC19A3, ADAMTS1, ICAM4, SRPX, GNG11, VGLL3, PPARG, MAOA, LAMP3, CA2, C7, FBLN5, BCHE, AQP1, MS4A2, S1PR1, TRHDE, CNTN6, CD52, CDH5, SLC16A4, CAV2, CLDN5, S100A4, HYAL1, FCN3, HLF, KCNJ15, JAM2, CPM, WISP2, SLC1A1, PTGDS, HIGD1B, F3, S100A14, CD93, FMO3, CACNA2D2, ZFPM2, STX11, CFP, CD36, RETN, RASSF9, RGN, S100P, DPP4, SPARCL1, SGCG, BMP2, SOCS2, S100A10, ALOX5AP, ITGAM, WFDC1, FHL1, CCDC68, SELE, IL33, TFPI, ANKRD1, LPL, TRPC6, MARCO, CD55, CXCL3, ADRB2, PIGR, ROS1, FOXF1, CST6, PROS1, PDK4, GSTA1, CDO1, ATP1A2, FAM107A, S100A12, FBP1, YAP1, IL6, ANG, SLCO2A1, TGFBR3, TREM1, VSIG4, PLSCR4, CFH, EMP1, P2RY1, ALDH2, PCOLCE2, FCN1, CTSH, TFPI2, MUC1, ABCG2

*DEGs, differentially expressed genes.

### 3.2 Biological function analysis and pathway enrichment analysis

GO analysis focused on “positive regulation of gene expression,” “positive regulation of transcription from RNA polymerase II promoter,” “cell division,” and “negative regulation of transcription from RNA polymerase II promoter” for biological process (BP) annotation. Moreover, it was abundant in the “extracellular space,” “extracellular region,” “plasma membrane,” “cytoplasm,” “nucleus,” and “nucleoplasm,” according to the cellular component (CC) annotation. In molecular function (MF) annotation, “protein binding,” “identical protein binding,” and “DNA binding” were clustered (Table 3). DEGs are primarily enriched in “Cell cycle,” “Complement and coagulation cascades,” and “Human T-cell leukemia virus 1 infection” in the KEGG pathway (Table 4).

**Table 3:** GO analysis

Category	Term	Counts	Ratio	*P* value
BP^*^	0010628∼positive regulation of gene expression	20	5.62	0.003
	0045944∼positive regulation of transcription from RNA polymerase II promoter	43	12.08	4.08 × 10^−5^
	0051301∼cell division	25	7.02	1.67 × 10^−7^
	0000122∼negative regulation of transcription from RNA polymerase II promoter	33	9.02	8.90 × 10^−4^
CC^1^	0005615∼extracellular space	83	23.31	8.24 × 10^−15^
	0005576∼extracellular region	81	22.75	9.85 × 10^−12^
	0005886∼plasma membrane	123	34.55	1.70 × 10^−5^
	0005737∼cytoplasm	124	34.83	7.86 × 10^−4^
	0005634∼nucleus	127	35.67	0.004
	0005654∼nucleoplasm	87	24.44	0.010
MF^2^	0005515∼protein binding	272	76.40	1.95 × 10^−6^
	0042802∼identical protein binding	52	14.61	4.39 × 10^−4^
	0003677∼DNA binding	40	11.24	0.003

*BP, Biology Process; CC^1^, Cellular Component; MF^2^, Molecular Function. Term: Term is the basic unit of GO, and each term corresponds to a GO class name, which is an attribute. Counts: The total number of genes enriched in this Go term. Ratio: Ratio represents the proportion of genes enriched in the Go term to the total genes. *P* value: *P* value represents significance, and *P* value < 0.05 is considered to be statistically significant for genes enriched in this term.

**Table 4:** KEGG pathway enrichment analysis

Category	Term	Counts	Ratio	*P* value
KEGG	04110: Cell cycle	17	4.78	6.62 × 10^−8^
	04610: Complement and coagulation cascades	11	3.09	4.10 × 10^−5^
_	05166: Human T-cell leukemia virus 1 infection	10	2.81	0.097

Term: Category of the pathway in KEGG pathway enrichment analysis. Counts: The total number of genes enriched in the pathway. Ratio: The proportion of genes enriched in the pathway to the total genes. *P* value: Significance enriched to pathways. *P* value < 0.05 was considered to be statistically significant for genes enriched in the pathway. From large to small, the degree of enrichment becomes more and more significant.

### 3.3 Construction of protein network and selection of hub gene

**Figure 4:**
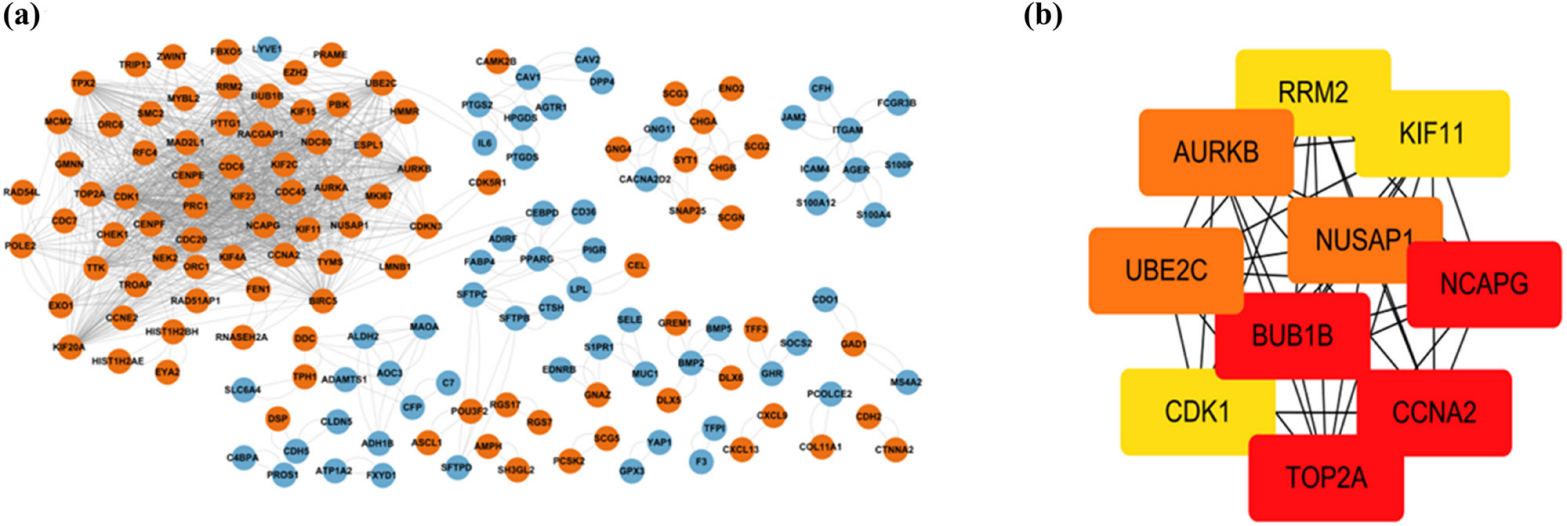
The construction of PPI network and significant gene modules analysis. (a) The PPI networks of differentially expressed genes and (b) the top ten genes in the PPI networks. The orange nodes represented upregulated genes, while the blue ones represented downregulated genes.

Blue nodes represent downregulated genes, whereas orange nodes stand for upregulated genes (Figure 4a and b). A total of 156 genes or nodes and 1,420 edges were enriched in the network. NCAPG, BUB1B, TOP2A, CCNA2, NUSAP1, UBE2C, AURKB, RRM2, CDK1, and KIF11 were the top ten hub genes (Figure 4b). Besides, all the parameters were set by default in the CytoHubba.

### 3.4 Screening for existing drugs that target the ten genes

Ten hub genes were matched with existing drugs using the drug–gene interaction module in the QuartataWeb database. Only two genes, TOP2A and RRM2, were found and matched to ten estimated medicinal drugs (Teniposide, Etoposide, Daunorubicin, Doxorubicin, Amrubicin, Dactinomycin, Epirubicin, Idarubicin, Cladribine, and Gallium nitrate) (Table 5). Screening criteria were: drug–gene interaction cut-off *p* < 0.05 and support from previous literature (Table 6).

**Table 5:** Significant drugs targeting hub genes

Gene	Drug ID	Drug name	Drug type	Drug group	*P* value
TOP2A	DB00445	Epirubicin	Small molecule drug	Approved	5.74 × 10^−3^
	DB01177	Idarubicin	Small molecule drug	Approved	6.46 × 10^−3^
	DB00997	Doxorubicin	Small molecule drug	Approved	5.17 × 10^−3^
	DB00970	Dactinomycin	Small molecule drug	Approved; investigational	5.44 × 10^−3^
	DB00773	Etoposide	Small molecule drug	Approved	4.49 × 10^−3^
	DB00444	Teniposide	Small molecule drug	Approved	3.45 × 10^−3^
	DB00694	Daunorubicin	Small molecule drug	Approved	4.92 × 10^−3^
	DB06263	Amrubicin	Small molecule drug	Approved; investigational	5.17 × 10^−3^
RRM2	DB00242	Cladribine	Small molecule drug	Approved; investigational	1.65 × 10^−2^
	DB05260	Gallium nitrate	Small molecule drug	Approved; investigational	7.64 × 10^−3^

**Table 6:** Publications related to the effective drugs targeted hub genes

Gene names	Drug name	Publications	
		Molecular or cellular level	Human body level
TOP2A	Epirubicin	[23]	[24]
	Idarubicin	[25]	
	Doxorubicin	[26]	[27]
			[28]
	Dactinomycin	[29]	
	Etoposide	[30]	[31]
			[32]
	Teniposide		[33]
	Daunorubicin	[34,35]	
	Amrubicin		[36]
RRM2	Cladribine	[37]	
	Gallium nitrate	[38,39]	

### 3.5 Validation of gene expression in SCLC

**Figure 5:**
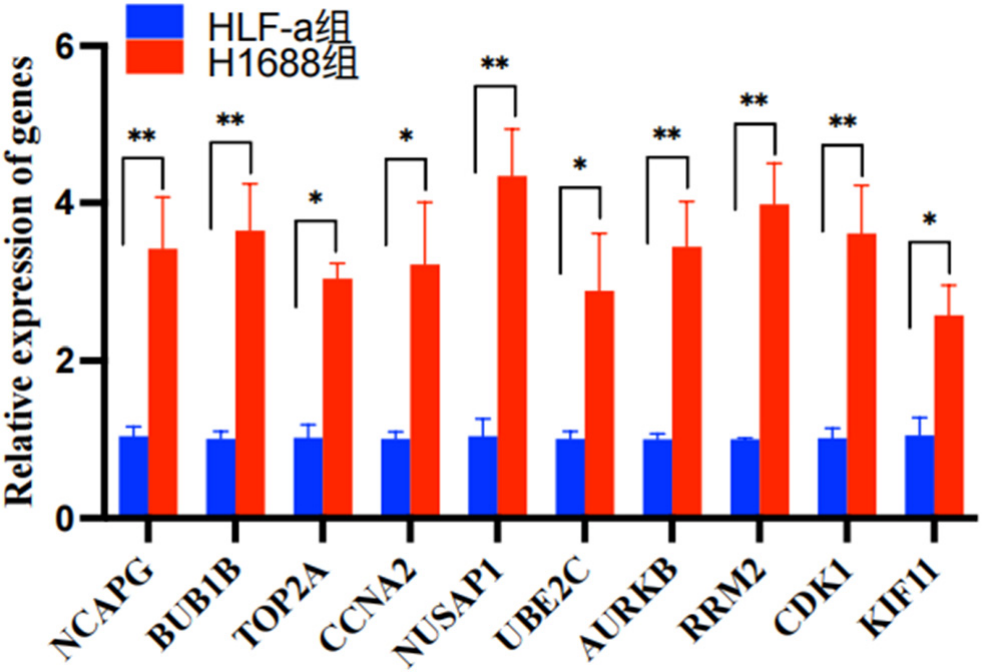
qPCR validation of hub genes in the normal human cell lines (HLF-a) and tumor cell lines (NCI-H1688). *P < 0.05. **P < 0.01.

With the aim of verifying the expression levels of NCAPG, BUB1B, TOP2A, CCNA2, NUSAP1, UBE2C, AURKB, RRM2, CDK1, and KIF11, normal lung cell lines and SCLC cell lines were selected. Additionally, the qRT-PCR assays were used with the purpose of quantifying the relative mRNA expression of the above genes in normal lung and SCLC cell lines. Based on the obtained findings, the mRNA expressions of the above genes in SCLC cell lines were greater in relative to those in normal lung cell lines (*P* < 0.05, Figure 5).

## 4 Discussion

Oncologists still face significant difficulties in treating SCLC owing to their high mutation rates and other clinical limitations. Patients with SCLC have low survival rates. However, during the past few decades, research on novel therapeutic strategies for treating SCLC has been limited [40]. Hence, there is an urgent need to identify target genes that can specifically and effectively target SCLC and thus correctly treat it. The development of high-throughput techniques and sophisticated computational tools has enabled the identification of relatively few genes that are characteristically deregulated in a given cancer cell among the thousands of normally expressed genes [41]. These methods offer novel approaches to diagnosis and treatment. Our study is the first to use bioinformatics to identify ten previously approved drugs that are related to RRM2 and TOP2A. Our findings might provide patients with SCLC new therapy options.

The current study analyzed GSE149507, GSE6044, and GSE30219 using the limma package and screened 195 upregulated and 167 downregulated genes. In the BP annotation in GO analysis, these genes were mostly enriched in “positive regulation of gene expression” and “cell division,” which are closely related to gene expression.” The DEGs in the CC category were mainly related to “extracellular space,” “extracellular region,” “plasma membrane,” “nucleus,” and “nucleoplasm,” and they showed close relationship to the extracellular and nuclear microenvironment. Additionally, the DEGs in the MF class were enriched for “protein binding” and “identical protein binding” terms and showed tight association with protein synthesis. The findings of GO analysis demonstrated that SCLC may play a pathogenic role through gene expression and translation in cells. DEGs were primarily enriched in cell cycle-related pathways including “Cell cycle” and “Complement and coagulation cascades” in the KEGG pathway. According to recent research, the cell cycle pathway makes a vital impact on developing SCLC [42]. Our results are in consistence with those of earlier research.

In the PPI network, 156 genes or nodes and 1,420 edges were enriched. We chose the top ten hub genes with the CytoHubba plugin in the Cytoscape software. This suggests that overexpression of NCAPG, BUB1B, TOP2A, CCNA2, NUSAP1, UBE2C, AURKB, RRM2, CDK1, and KIF11 may promote SCLC progression.

Additionally, to assess the level of ten hub genes in SCLC cells and healthy human lung cells, the validation of the qRT-PCR assay was performed. Moreover, the obtained findings demonstrated that similar gene expression trends of 10 hub genes in SCLC cells and normal human lung cells were demonstrated by qPCR, verifying the accuracy of our findings.

TOP2A produces polyisomerase II (TOPII), a crucial enzyme that alters the DNA topology by joining two double-stranded DNA molecules. TOPII is essential for gene transcription and replication. The aberrant expression of TOP2A can be related to poor prognosis in the lung, esophageal, breast, ovarian, and oral cancers [43]. A previous study showed that TOP2A is engaged in the occurrence and development of SCLC through inhibiting ectopic expression of miR-27a-5p and miR-34b-3p [44]. Therefore, TOP2A may show close relationship to the occurrence and prognosis of SCLC through comprehensive analysis.

AURKB, a serine/threonine protein kinase, is a crucial mitotic regulator. The oncogenic properties of AURKB have been studied in various tumors [45]. According to a recent study, SCLCs lacking the RB1 tumor suppressor gene are overly relied on Aurora B kinase for survival. Patients with SCLC typically have RB1 gene mutations. Furthermore, the study found that Aurora B kinase exerts a role in suppressing tumor cell growth in multiple SCLC models [46].

BUB1B, a member of the spindle assembly checkpoint protein family, is necessary for the anaphase of mitosis. Multiple research works have confirmed that abnormal BUB1B expression is related to tumor prognosis [47]. A large-scale analysis of the transcriptional profile of NSCLC suggested that BUB1B is a hub gene in adenocarcinoma (ADC, lung adenocarcinomas) [48]. Thus, BUB1B is a promising candidate gene.

The cell cycle regulator Cyclin-A2 (CCNA2) regulates mitotic G1/S and G2/M phases [49]. The occurrence and development of tumors may be caused by impaired regulation of this process [50]. In addition, CCNA2 is abnormally expressed in other tumors [51].

RRM2, the ribonucleoside-diphosphate reductase subunit M2B, has been identified as a gene with poor survival prognosis through network analysis and multivariate prognostic analysis in patients with LUAD [52]. Bioinformatics analysis by Chen et al. [53] identified RRM2 as the hub gene for SCLC.

UBE2C, a cell cycle-regulated ubiquitin ligase, regulates mitosis. Some researchers have reported that UBE2C shows close relationship to tumor occurrence, proliferation, and other behaviors [54]. Additionally, Wang et al. [55] discovered that UBE2C is tightly correlated with angiogenesis in NSCLC, confirming the speculation of previous studies.

Cyclin-dependent kinase 1 (CDK1) binds to cyclin B1 (CCNB1) or cyclin B2 (CCNB2) to form a complex that regulates the mitotic initiation process. Its dysregulation has been indicated to correlate with tumor cell proliferation [56]. A bioinformatics study revealed that CDK1 stimulates the stemness of lung cancer cells by the interaction with SOX2 and that increased CDK1 expression shows relationship to lower overall survival in patients suffering from lung cancer. Therefore, CDK1 may play the role of a potential biomarker [57].

Similarly, NUSAP1 and NCAPG stimulate the progression of NSCLC by controlling the BTG2/PI3K/Akt signaling pathway and upregulating LGALS1 expression [58,59]. They are also highly expressed in various tumors [60,61].

The current work used bioinformatics methods to screen FDA-approved drugs and reposition them as new anticancer drugs. Our study showed that TOP2A and RRM2 matched predicted FDA-approved drugs.

The TOP2A gene matched with eight drugs, and they are adopted for cancer therapy, among the matches between the input hub genes and the selected drugs. Etoposide, a semisynthetic derivative of podophyllotoxin with antitumor activity, was chosen as the first-line chemotherapy for SCLC among these drugs [31,32,62]. Teniposide refers to a cytotoxic drug used to treat refractory childhood acute lymphoblastic leukemia [63]. Epirubicin is an anthracycline antineoplastic drug used as adjuvant therapy after primary breast cancer resection [64]. Idarubicin is also an anthracycline antineoplastic drug, and its indications are adult acute myeloid leukemia [65]. Doxorubicin is an anthracycline antibiotic that is cytotoxic. It has a wide range of indications and can be used to treat various cancers [66]. Valrubicin is a chemotherapeutic drug which can be adopted for treating bladder cancer [67]. Daunorubicin is an anthracycline aminoglycoside antitumor drug used to induce remission in adults with acute non-lymphocytic leukemia and children and adults with acute lymphoblastic leukemia [68]. Amrubicin, an anthracycline, is currently being studied for SCLC treatment [36].

RRM2 matches only the two FDA-approved drugs. Cladribine is a purine analog and antineoplastic agent used to treat adults with highly active relapsing multiple sclerosis [69]. Gallium nitrate is used to treat cancer-related hypercalcemia and non-Hodgkin lymphoma [70].

In conclusion, NCAPG, BUB1B, TOP2A, CCNA2, NUSAP1, UBE2C, AURKB, RRM2, CDK1, and KIF11 are potential markers for diagnosing and treating SCLC. Additionally, we selected and constructed two genes, TOP2A and RRM2, as well as their potential related drugs to offer novel ideas for treating SCLC. Moreover, our experiments were subject to significant bias. Our shortcoming was that we did not validate this through relevant experiments. Therefore, these drugs require validation using relevant experimental models.
